# Supplementary material for: Meaning Making as a Lifebuoy in Dementia Caregiving: Predicting Depression from a Generation Perspective Using a Fuzzy-Set Qualitative Comparative Analysis
Source: Int J Environ Res Public Health. 2022 Nov 25;19(23):15711. doi: 10.3390/ijerph192315711 (PMC9736359; doi:10.3390/ijerph192315711)
Supplement: Supplementary file 1 [file ijerph-19-15711-s001.zip › ijerph-1940921-supplementary.pdf]

Table S1. An example of contrarian case analysis for depressive symptoms at follow-up and a sense of loss/powerlessness.

			**Percentile Group of Depressive symptoms at follow-up**					
			**1**	**2**	**3**	**4**	**5**	**Total**
**Percentile Group of Sense of loss/ powerlessness** (phi^2 = 0.69, $p < 0.001$)	**1**	Count	20	10	6	4	1	41
		(%)	48.8%	24.4%	14.6%	9.8%	2.4%	100.0%
	**2**	Count	20	7	8	8	3	46
		(%)	43.5%	15.2%	17.4%	17.4%	6.5%	100.0%
	**3**	Count	4	3	14	2	4	27
		(%)	14.8%	11.1%	51.9%	7.4%	14.8%	100.0%
	**4**	Count	2	2	9	9	6	28
		(%)	7.1%	7.1%	32.1%	32.1%	21.4%	100.0%
	**5**	Count	0	1	4	6	14	25
		(%)	0.0%	4.0%	16.0%	24.0%	56.0%	100.0%
	**Total**	Count	46	23	41	29	28	167
		(%)	27.5%	13.8%	24.6%	17.4%	16.8%	100.0%

Note. Numbers in (%) are within the percentile group of sense of loss/powerlessness. Numbers in bold boxes are the contrarian case numbers.

Table S2. Reliability and correlation matrix.

Variable	Cronbach's Alpha	*M (SD)*	1	2	3	4	5	6	7	8	9	10
1. Depressive symptoms at follow-up	0.915	4.86 (5.37)	-									
2. Generation	-	-	−0.119	-								
3. Sense of loss/ powerlessness	0.888	57.31 (9.87)	0.493 ***	−0.201 **	-							
4. Sense of provisional meaning	0.931	70.81 (9.36)	−0.030	0.069	−0.051	-						
5. Caregiver burden	0.763	11.16 (2.90)	0.422 ***	−0.210 **	0.619 ***	−0.104	-					
6. Depressive symptoms at baseline	0.866	3.46 (4.05)	0.516 ***	−0.005	0.500 ***	−0.180 *	0.443 ***	-				
7. Caregiver distress	0.900	13.71 (9.82)	0.397 ***	−0.070	0.403 ***	−0.011	0.456 ***	0.428 ***	-			
8. Resilience	0.923	35.22 (6.58)	−0.238 **	−0.159	−0.297 ***	0.464 ***	−0.310 ***	−0.359 ***	−0.179 *	-		
9. Adaptation	-	-	0.284 ***	−0.079	0.157*	0.046	0.258 ***	0.163*	0.386 ***	−0.102	-	
10. NPS severity	0.855	11.60 (6.77)	0.328 ***	−0.015	0.321 ***	0.018	0.390 ***	0.377 ***	0.840 ***	−0.166 *	0.331 ***	-

Note. *M* and *SD* are used to represent mean and standard deviation, respectively. $^{*}p < 0.05$, $^{**}p < 0.01$, $^{***}p < 0.001$. NPS = Neuropsychiatric Symptoms.
